# Epidemiology of Canine *Wei Syndrome* and Its Hemorheology Characteristics

**DOI:** 10.3390/ani14182658

**Published:** 2024-09-12

**Authors:** Shuo Yang, Yuting Liu, Bingjie Chen, Jie Mi, Xiangbo Tai, Wuren Ma

**Affiliations:** 1 College of Veterinary Medicine, Northwest A&F University, Yangling 712100, China; yangshuo123@nwafu.edu.cn (S.Y.); lengyu_s7@nwafu.edu.cn (Y.L.); 2 Xi’an Veterinary Teaching Hospital, Northwest A&F University, Xi’an 710065, China; chenbingjie8911@163.com (B.C.); mjmj0625@163.com (J.M.); tai1009971821@163.com (X.T.); 3 Institute of Traditional Chinese Veterinary Medicine, Northwest A&F University, Yangling 712100, China

**Keywords:** dogs, paraplegia, blood rheology, pathogenesis

## Abstract

**Simple Summary:**

In Traditional Chinese Medicine and Traditional Chinese Veterinary Medicine, *Wei Syndrome* is a prevalent condition. Canine *Wei Syndrome* usually exhibits symptoms including hind limb paralysis, muscle weakness or even atrophy, disappearance of deep or shallow pain, and urinary and fecal incontinence. Hemorheological indicators play a crucial role in assessing blood viscosity. However, research on canine *Wei Syndrome* and its hemorheological characteristics remains limited. Understanding the factors such as gender, season, breed, and age that predispose dogs to *Wei Syndrome* can aid in its prevention. Early detection and diagnosis of canine *Wei Syndrome* can be facilitated by monitoring hemorheological data. Therefore, investigating the epidemiology of canine *Wei Syndrome* and its correlation with hemorheology is of paramount importance. This study revealed that male Poodle dogs aged 3–6 years are more susceptible to *Wei Syndrome* during Winter, with abnormal hemorheology being a distinct feature among affected dogs. These findings contribute significantly to the prevention and management of canine *Wei Syndrome* and offer valuable insights for the treatment of dogs with *Wei Syndrome*.

**Abstract:**

Canine paraplegia is a common condition in small animal medicine, referred to as *Wei Syndrome* (*WS*) in Traditional Chinese Veterinary Medicine (TCVM). Common clinical manifestations encompass hind limb paralysis, motor dysfunction, muscle atrophy, and the absence of pain perception. *WS* is considered a difficult-to-treat disease in small animal practice. The objective of this study was to investigate the epidemiology of canine *WS* and the characteristics of hemorheology. A total of 53 dogs with *WS* and 53 healthy dogs were included in this study. A retrospective case-controlled study design was employed. Data regarding the gender, season of *WS* occurrence, breed, and age of dogs with *WS*, as well as hemorheology from dogs with *WS* and healthy dogs, were collected and analyzed using SPSS 27.0. The study findings revealed that male dogs were more susceptible to *WS* (77.36%, 41/53). *WS* cases occurred more frequently in Winter (33.96%, 18/53), and were commonly found in Poodle breeds (43.40%, 23/53). The most affected age of *WS* was between 3 and 6 years old (54.72%, 29/53). Except for plasma viscosity and fibrinogen, the hemorheology indices of canine *WS* were significantly higher than those of healthy dogs (*p* < 0.05), especially in male dogs, Poodles and Bulldogs, those between 3 to 10 years, and in Autumn and Winter. This study provides evidence that male Poodles and Bulldogs aged 3 to 6 years are more prone to developing *WS*, with Winter being the season of high disease incidence. Abnormal hemorheology is a characteristic feature in dogs with *WS*, which should be considered during the treatment of *WS*.

## 1. Introduction

Hind limb weakness, and even paraplegia, is a common clinical sign in dogs in small animal medicine. This condition is referred to as *Wei Syndrome* (*WS*) in Traditional Chinese Veterinary Medicine (TCVM) and is caused by external or internal pathogenic factors of the body [1]. In TCVM, *WS* is considered to be caused by a lack of certain nutrients in the body, leading to inadequate nourishment of the skin, muscles, tendons, sinews, and joints [2]. The main clinical symptoms of *WS* include tendon relaxation, limb weakness, limited mobility, and muscle atrophy, which can progress to paraplegia. Due to the fact that in the Traditional Chinese Medicine (TCM) system, the pathogenesis of *WS* is related to the four organs of the liver, spleen, lungs, and kidneys, relevant clinical symptoms may occur. These include dry eyes, irritability, decreased appetite, loose or dry stool, swollen limbs, coarse coat, red rashes, dry cough with less phlegm, thirst, urinary and fecal incontinence, and abnormal estrus cycle [1].

According to TCM theory, although there is no specific term for intervertebral disc degeneration, it is posited that this condition is associated with *WS* [3]. The clinical manifestations of *WS* and intervertebral disc degeneration are analogous, and TCM modalities such as acupuncture and moxibustion are employed in the treatment of canine intervertebral disc diseases [1,4]. Consequently, it is reasonable to consider *WS* and intervertebral disc disease as the same syndrome in TCVM and western medicine, respectively. In TCVM theory, *Qi* plays a role in blood circulation, and deficiency of *Qi* can lead to blood stasis, which is the western medicine term for thrombus formation. As a result, microcirculation disorders can occur. Studies have shown that thrombus formation can contribute to the development of paraplegia in dogs [5].

We hypothesize that increased blood viscosity may be associated with an elevated risk of paraplegia in dogs, resulting in an abnormal microcirculatory environment. However, the hemorheological characteristics of canine *WS* have not yet been investigated. Therefore, this study aimed to investigate the hemorheological profiles of dogs presenting with paraplegia at the Xi’an Veterinary Teaching Hospital of the Northwest A&F University to explore their distinctive features.

## 2. Materials and Methods

### 2.1. Case Selection Criteria

A retrospective review was performed on the medical records of dogs diagnosed with *WS* at the Xi’an Veterinary Teaching Hospital of Northwest A&F University from April 2020 to March 2023. The *WS* group consisted of dogs exhibiting symptoms such as muscle relaxation, weakness, muscle atrophy, weak or loss of deep sensation, and inability to move freely. The control group comprises healthy individuals who have not exhibited symptoms of paralysis at the animal shelter, which were considered healthy after physical examination.

### 2.2. Hemorheology

A 5 mL blood sample was taken and placed in a lithium heparin anticoagulant tube. A fully automatic blood viscosity dynamic analyzer (Chongqing Nanfang Numerical Control Equipment Co., Ltd., Chongqing, China) was used to measure hemorheology. After the temperature rose to 37 °C, the sample was placed in any aperture, the corresponding aperture was selected on the computer, and “measure whole blood” was clicked. Once the test was completed, the sample was removed and placed in a centrifuge (Hunan Xiangyi Laboratory Instrument Development Co., Ltd., Changsha, China) and centrifuged for 5 min at 4500 rpm. After centrifugation, the sample was returned to the original aperture, “measure plasma” was clicked, and once the test was finished, the sample was removed, and the testing was completed.

### 2.3. Clinical Data

The medical records of each case were reviewed, and the following information was collected: gender, visiting time, breed, age of patient, hemorheology test results. Gender and hemorheology data were collected for control group.

### 2.4. Data Analysis

Epidemiological survey results are expressed as percentages. All parameters in the analysis of hemorheology characteristics were represented by mean ± standard deviation (Mean ± SD). According to the normality of the data distribution, *t*-test or Mann-Whitney rank sum test is used. Data analysis was conducted using SPSS 27.0 (SPSS Inc., Chicago, IL, USA) statistical analysis software, with *p* < 0.05 showing significance.

## 3. Results

### 3.1. Epidemiology of Canine Wei Syndrome

**Figure 1:**
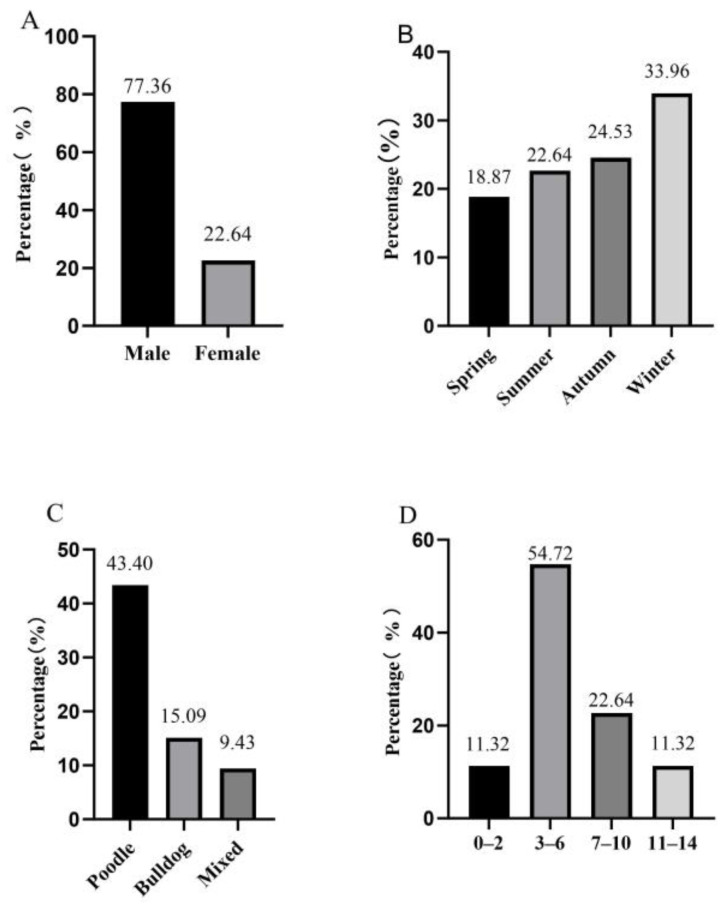
Epidemiology of *WS* dogs. (**A**) Gender difference; (**B**) Seasonal difference; (**C**) Breed difference; (**D**) Age difference.

Gender Distribution: Among the sampled dogs, male dogs accounted for 77.36% (41/53) while females accounted for 22.64% (12/53), see Figure 1A.

Seasonal Occurrence: The occurrence of *WS* varied across different seasons. Winter is the season with the highest occurrence of *WS*, accounting for 33.96% (18/53) of the cases. This was followed by Autumn with 24.53% (13/53), Summer with 22.64% (12/53), and Spring with 18.87% (10/53), see Figure 1B.

Breed Predisposition: Poodles were found to be the most prone breed for *WS*, accounting for 43.40% (23/53) of the cases. This was followed by Bulldogs with 15.09% (8/53) and mixed-breed dogs with 9.43% (5/53), see Figure 1C.

Age Distribution: The mean age of dogs with *WS* was 5.8 ± 3.3 years old. The youngest patient was 10 months old, while the oldest patient was 14 years old. Dogs in the age range of 3–6 years old were overrepresented in *WS*, accounting for 54.72% (29/53) of the cases. This was followed by dogs in the age ranges of 7–10 years old (22.64%, 12/53), 11–14 years old (11.32%, 6/53), and 0–2 years old (11.32%, 6/53), see Figure 1D.

### 3.2. Hemorheology Characteristics of Canine WS

#### 3.2.1. General Hemorheology Characteristics of Canine WS

There were significant differences in various blood viscosity measurements and other related parameters between the *WS* group and the control group. Specifically, the *WS* group exhibited higher values for whole blood high shear viscosity at 200·S^−1^ (Hŋb 200·S^−1^, *p* < 0.001), whole blood medium shear viscosity at 30·S^−1^ (Mŋb 30·S^−1^, *p* < 0.001), whole blood low shear viscosity at 1·S^−1^ (Lŋb 1·S^−1^, *p* < 0.001), whole blood high shear reduced viscosity at 200·S^−1^ (Hŋr 200·S^−1^, *p* < 0.001), whole blood medium shear reduced viscosity at 30·S^−1^ (Mŋr 30·S^−1^, *p* < 0.001), whole blood low shear reduced viscosity at 1·S^−1^ (Lŋr 1·S^−1^, *p* < 0.001), erythrocyte deformation index (EDI, *p* < 0.001), high shear flow resistance (HFR, *p* < 0.001), medium shear flow resistance (MFR, *p* < 0.001), low shear flow resistance (LFR, *p* < 0.001), erythrocyte aggregation index (EAI, *p* < 0.01), Carson viscosity (QwX, *p* < 0.001), Carson yield stress (CS, *p* < 0.001), erythrocyte aggregation coefficient (VAI, *p* < 0.01), erythrocyte rigidity index (ERI, *p* < 0.001), and hematocrit (Hct, *p* < 0.001). Conversely, there were significant differences in plasma viscosity (ŋp, *p* < 0.001) and fibrinogen (Fb, *p* < 0.001) between the *WS* group and the control group, with lower values observed in the *WS* group when compared to the control group. These findings are summarized in Table 1.

#### 3.2.2. Hemorheology Characteristics of Canine *WS* on Gender

The Hŋb 200·S^−1^ (*p* < 0.001), Mŋb 30·S^−1^ (*p* < 0.001), Lŋb 1·S^−1^ (*p* < 0.01), Hŋr 200·S^−1^ (*p* < 0.001), Mŋr 30·S^−1^ (*p* < 0.001), Lŋr 1·S^−1^ (*p* < 0.001), EDI (*p* < 0.001), HFR (*p* < 0.001), MFR (*p* < 0.001), LFR (*p* < 0.01), EAI (*p* < 0.01), QwX (*p* < 0.01), CS (*p* < 0.001), VAI (*p* < 0.01), ERI (*p* < 0.001), and Hct (*p* < 0.001) in the male *WS* group were significantly higher than those in the male control group. The ŋp (*p* < 0.001) and Fb (*p* < 0.001) in the male *WS* group were significantly lower than those in the male control group.

The Lŋb 1·S^−1^ (*p* < 0.05), Hŋr 200·S^−1^ (*p* < 0.01), Mŋr 30·S^−1^ (*p* < 0.01), Lŋr 1·S^−1^ (*p* < 0.01), EDI (*p* < 0.05), LFR (*p* < 0.05), ERI (*p* < 0.01), and Hct (*p* < 0.05) in the female *WS* group were significantly higher than those in the female control group. The ŋp (*p* < 0.05) and Fb (*p* < 0.05) in the female *WS* group were significantly lower than those in the female control group, as shown in Table 2.

#### 3.2.3. Hemorheology Characteristics of Canine *WS* on Season

The Hŋr 200·S^−1^ (*p* < 0.001), Mŋr 30·S^−1^ (*p* < 0.001), Lŋr 1·S^−1^ (*p* < 0.001), EDI (*p* < 0.001), CS (*p* < 0.05), Hct (*p* < 0.05), and ERI (*p* < 0.001) of the *WS* group in Spring were significantly higher than those of the control group, and ŋp (*p* < 0.001) and Fb (*p* < 0.001) were significantly lower than those in the control group.

The Hŋr 200·S^−1^ (*p* < 0.001), Mŋr 30·S^−1^ (*p* < 0.001), Lŋr 1·S^−1^ (*p* < 0.001), EDI (*p* < 0.001), Hct (*p* < 0.05), and ERI (*p* < 0.001) of *WS* group in Summer were significantly higher than those of the control group, and ŋp (*p* < 0.001) and Fb (*p* < 0.001) were significantly lower than those in the control group.

The Hŋb 200·S^−1^ (*p* < 0.01), Mŋb 30·S^−1^ (*p* < 0.01), Lŋb 1·S^−1^ (*p* < 0.05), Hŋr 200·S^−1^ (*p* < 0.001), Mŋr 30·S^−1^ (*p* < 0.001), Lŋr 1·S^−1^ (*p* < 0.001), EAI (*p* < 0.05), HFR (*p* < 0.01), MFR (*p* < 0.01), LFR (*p* < 0.01), EDI (*p* < 0.001), QwX (*p* < 0.01), CS (*p* < 0.01), VAI (*p* < 0.05), ERI (*p* < 0.001), and Hct (*p* < 0.01) of the *WS* group in Autumn were significantly higher than those of the control group. The ŋp (*p* < 0.001) and Fb (*p* < 0.001) of *WS* group in Autumn were significantly lower than those of the control group.

The Hŋb 200·S^−1^ (*p* < 0.01), Mŋb 30·S^−1^ (*p* < 0.01), Lŋb 1·S^−1^ (*p* < 0.01), Hŋr 200·S^−1^ (*p* < 0.001), Mŋr 30·S^−1^ (*p* < 0.001), Lŋr 1·S^−1^ (*p* < 0.001), EAI (*p* < 0.05), HFR (*p* < 0.01), MFR (*p* < 0.01), LFR (*p* < 0.01), EDI (*p* < 0.001), QwX (*p* < 0.01), CS (*p* < 0.01), VAI (*p* < 0.05), ERI (*p* < 0.001), and Hct (*p* < 0.01) of the *WS* group in Winter were significantly higher than those of the control group. The ŋp (*p* < 0.001) and Fb (*p* < 0.001) of *WS* group in Winter were significantly lower than those of the control group, as shown in Table 3.

#### 3.2.4. Hemorheology Characteristics of Canine *WS* on Breed

The Hŋb 200·S^−1^ (*p* < 0.001), Mŋb 30·S^−1^ (*p* < 0.001), Lŋb 1·S^−1^ (*p* < 0.001), Hŋr 200·S^−1^ (*p* < 0.001), Mŋr 30·S^−1^ (*p* < 0.001), Lŋr 1·S^−1^ (*p* < 0.001), EAI (*p* < 0.001), HFR (*p* < 0.001), MFR (*p* < 0.001), LFR (*p* < 0.001), EDI (*p* < 0.001), QwX (*p* < 0.001), CS (*p* < 0.001), VAI (*p* < 0.001), ERI (*p* < 0.001), and Hct (*p* < 0.001) in Poodles in the *WS* group were significantly higher than those of the control group. The ŋp (*p* < 0.001) and Fb (*p* < 0.001) of Poodles in the *WS* group were significantly lower than those of the control group.

The Hŋb 200·S^−1^ (*p* < 0.01), Mŋb 30·S^−1^ (*p* < 0.01), Lŋb 1·S^−1^ (*p* < 0.01), Hŋr 200·S^−1^ (*p* < 0.001), Mŋr 30·S^−1^ (*p* < 0.01), Lŋr 1·S^−1^ (*p* < 0.01), EAI (*p* < 0.01), HFR (*p* < 0.01), MFR (*p* < 0.01), LFR (*p* < 0.01), EDI (*p* < 0.001), QwX (*p* < 0.01), CS (*p* < 0.01), VAI (*p* < 0.01), ERI (*p* < 0.001), and Hct (*p* < 0.001) in Bulldogs in the *WS* group were significantly higher than those of the control group. The ŋp (*p* < 0.01) and Fb (*p* < 0.01) of in Bulldogs in the *WS* group were significantly lower than those of the control group.

The Hŋr 200·S^−1^ (*p* < 0.01), Mŋr 30·S^−1^ (*p* < 0.01), Lŋr 1·S^−1^ (*p* < 0.01), EAI (*p* < 0.05), EDI (*p* < 0.05), CS (*p* < 0.05), VAI (*p* < 0.05), and ERI (*p* < 0.01) of the mixed breed group with *WS* were significantly higher than those of the control group, as shown in Table 4. The ŋp (*p* < 0.05) and Fb (*p* < 0.05) of the mixed breed group with *WS* were significantly lower than those of the control group.

#### 3.2.5. Hemorheology Characteristics of Canine *WS* on Age

The age of dogs was classified into 4 groups: 0–2 years old group, 3–6 years old group, 7–10 years old group, and 11–14 years old group.

In the 0–2 years old group, the Hŋr 200·S^−1^ (*p* < 0.001), Mŋr 30·S^−1^ (*p* < 0.001), Lŋr 1·S^−1^ (*p* < 0.001), EAI (*p* < 0.01), EDI (*p* < 0.01), CS (*p* < 0.05),VAI (*p* < 0.01), ERI (*p* < 0.001), and Hct (*p* < 0.05) of dogs with *WS* were significantly higher than that of the control group. In addition, there were significant differences in ŋp (*p* < 0.01) and Fb (*p* < 0.01) between the *WS* group and the control group, both of which were lower than the control group.

In the 3–6 years old group, the Hŋb 200·S^−1^ (*p* < 0.01), Mŋb 30·S^−1^ (*p* < 0.01), Lŋb 1·S^−1^ (*p* < 0.05), Hŋr 200·S^−1^ (*p* < 0.001), Mŋr 30·S^−1^ (*p* < 0.001), Lŋr 1·S^−1^ (*p* < 0.001), EDI (*p* < 0.001), HFR (*p* < 0.01), MFR (*p* < 0.01), LFR (*p* < 0.01), EAI (*p* < 0.05), QwX (*p* < 0.01), CS (*p* < 0.01), VAI (*p* < 0.05), ERI (*p* < 0.001), and Hct (*p* < 0.001) of dogs with *WS* also showed significant differences compared to the control group, all of which were higher than the control group. The ŋp (*p* < 0.001) and Fb (*p* < 0.001) of dogs with *WS* were significantly lower than those of the control group.

In the 7–10 years old group, the Hŋb 200·S^−1^ (*p* < 0.001), Mŋb 30·S^−1^ (*p* < 0.001), Lŋb 1·S^−1^ (*p* < 0.001), Hŋr 200·S^−1^ (*p* < 0.001), Mŋr 30·S^−1^ (*p* < 0.001), Lŋr 1·S^−1^ (*p* < 0.001), EDI (*p* < 0.01), HFR (*p* < 0.001), MFR (*p* < 0.001), LFR (*p* < 0.001), EAI (*p* < 0.001), QwX (*p* < 0.001), CS (*p* < 0.001), VAI (*p* < 0.001), ERI (*p* < 0.001), and Hct (*p* < 0.01) of dogs with *WS* also showed significant differences compared to the control group, all of which were higher than the control group. The ŋp (*p* < 0.01) and Fb (*p* < 0.01) of dogs with *WS* were significantly lower than those of the control group.

There was no significant difference in all data between the group with *WS* aged 11–14 years old and control dogs, as shown in Table 5.

## 4. Discussion

Based on the collected data, it has been found that male dogs are more prone to *WS* compared to females, accounting for 77.36% of the cases. As to seasons, it was observed that Winter is the most susceptible season for *WS*, accounting for 33.96% of the cases, followed by Autumn. In terms of breed, Poodles showed the highest breed tendency towards *WS*, accounting for 43.40% of the cases, followed by Bulldogs and mixed-breed dogs. When considering the age of dogs, it was observed that dogs aged between 3 to 6 years old are the most prone to *WS*, accounting for 54.72% of the cases, followed by dogs aged 7 to 10 years old.

As to the hemorheology of the *WS* group, there were significant differences compared to those of control group. Overall, there was an increasing trend in these indexes. Furthermore, the changes in hemorheology differ among dogs based on their gender, seasons, breeds, and ages. However, it is important to note that there is limited research conducted on the relationship between canine *WS* and hemorheology.

*WS* refers to the inability of limbs to move and muscle atrophy. In small animal practice, it has been observed that the hind limbs of dogs are more commonly affected [1]. According to western medicine, the primary cause of paralysis is intervertebral disc disease, which occurs when the intervertebral disc’s nucleus pulposus degenerates and calcifies, causing it to protrude through the fibrous ring, which results in compression and contusion of the spinal cord [3,6]. In the field of medicine, it is well-established that paralysis can be a consequence of a stroke. Research by Wang C has shown that post-stroke patients exhibit changes in their hemorheology data, indicating a correlation between paralysis and alterations in hemorheology [7]. Therefore, a similar correlation between paralysis and hemorheology may exist in dogs as well. 

According to our results, it is suggested that males have a higher susceptibility to *WS*. The survey results by Xiao B [4] and Ma LS et al. [8] demonstrated that the probability of developing intervertebral disc disease in male dogs is 79.25% and 78%, respectively. In line with these findings, surveys conducted by Ferreira AJ [9] and Fenn J [10] indicated that the likelihood of intervertebral disc disease in males was 61% and 52.9%, respectively. In human medicine, studies indicated that the incidence rate of intervertebral disc diseases in young and middle-aged men is higher compared to women. However, in the elderly population, women are more prone to develop intervertebral disc diseases than men [11]. The lack of estrogen can exacerbate intervertebral disc degeneration caused by spinal instability. On the other hand, estrogen supplementation can help reduce intervertebral disc degeneration associated with osteoporosis [12]. Additionally, research by Dorn M suggests that the risk of intervertebral disc herniation in sterilized female dogs is higher than in non-sterilized female dogs, implying that a decrease in estrogen levels may increase the incidence rate of intervertebral disc herniation in dogs [13]. Based on the findings from human medicine, estrogen levels appear to play a significant role in the pathogenesis of intervertebral disc disease. This suggests that estrogen could potentially be used as a therapeutic approach to prevent or treat intervertebral disc disease.

It was found that dogs with *WS* suffered the most in Winter. Xiao B’s study shows that temperature changes are significant from March to April, which is the peak of the incidence of intervertebral disc disease. The results from our study show a different pattern [4]. To explore the reasons behind this discrepancy and the potential relationship with different geographical areas, further research is needed. Nonetheless, other studies have indicated that lower ambient temperatures can contribute to increased pain and muscle damage, leading to a higher incidence of intervertebral disc disease in colder regions [14]. This indirectly supports the notion that *WS* is more likely to occur in areas with lower temperatures, such as Shaanxi. These findings reflect the reliability of the survey results.

The research has identified certain breeds that are more susceptible to *WS*, including Poodles, Bulldogs, and mixed-breed dogs. Studies conducted by Xiao B [4] and Ma LS [8] in China have found that Pekingese and Poodles are among the breeds most vulnerable to *WS*. Similarly, investigations by Ferreira AJ [9] and Fenn J [10] have identified Dachshunds, Cocker spaniels, mixed-breed dogs, Poodles, and Bulldogs as high-risk breeds. Interestingly, this investigation did not find Dachshunds, Cocker spaniels, and Pekingese to be at a higher risk for *WS*. This may be due to the different preferences for dog breeds and breeding practices in different regions. Chondrodysplasia breeds, such as French Bulldogs, Dachshunds, Pekingese, Beagles, and Poodles, have been determined to have a higher risk of intervertebral disc disease [15]. It is well known that purebred dogs can be more prone to certain diseases due to genetic factors. However, this study has also revealed that mixed-breed dogs are at a high risk for *WS*, which adds complexity to our understanding of the disease. Further investigation and research are necessary to comprehend why mixed-breed dogs are susceptible to *WS*.

In terms of age, dogs between the ages of 3 to 6 years old are most susceptible to *WS*. Ma LS’s investigation reported that most dogs with *WS* are aged between 3 and 8 [8], while Xiao B’s survey found the highest incidence between the ages of 5 to 6 (included 6-year-old) [4]. Ferreira AJ’s study identified the period around 5 years old as one of high incidence [9]. In Brisson BA’s study, the average age of dogs with intervertebral disk disease was also 5 years old [16]. While there is slight variation in the specific ages mentioned, all studies converge around the middle life stage, as defined by the AAHA canine life stage [17], which indicates that adult dogs are prone to developing *WS*. In human medicine, the incidence rates of intervertebral disc disease increase with age, affecting approximately 10% of men at the age of 50 and up to 50% at the age of 70 [18]. These findings suggest a common life stage susceptibility to intervertebral disc issues in both humans and dogs.

According to TCVM theory, *Qi* stagnation and blood stasis are implicated in the development of *WS*. Elevated red blood cell aggregation enhances local blood viscosity, diminishes local blood flow, and can precipitate thrombosis [19], which forms the pathological foundation of the blood stasis syndrome. In the context of *WS*, an increase in blood viscosity is anticipated.

Our investigation revealed that all hemorheology indexes significantly differed in the male *WS* group, whereas only 10 out of 18 hemorheology indexes in the female *WS* group showed a significant difference. *WS* can affect the blood viscosity of animals. It is known that erythrocytes, ŋp, and Fb are factors that positively correlate with blood viscosity. Interestingly, both the male *WS* group and the female *WS* group showed a decrease in ŋp and Fb levels. However, from the overall data of hemorheology, there are more indicators showing an upward trend than indicators showing a downward trend, and the ERI and Hct in the *WS* groups show an upward trend in both genders. In addition, the EAI and VAI of the male *WS* group also significantly increased. So, dogs with *WS* have higher blood viscosity. The deformability of red blood cells is one of the main determining factors of blood viscosity [20,21,22]. A study has found that female red blood cells have stronger deformability [23], as the concentration of estradiol in red blood cells can affect the deformability of red blood cells [24]. In our study, it was also shown that the EDI of female control group is greater than that of male control group. In Raberin A’s research, it was shown that male blood viscosity was higher than female blood viscosity at any shear rates [25]. Based on the above information, male dogs have higher blood viscosity than female dogs, and are more susceptible to *WS* than female dogs. Although ŋp and Fb show a decreasing trend after suffering from *WS*, from the observation of control groups, the ŋp and Fb of female healthy dogs are lower than those of males, indicating that males are more prone to *WS* than females.

In our study, we explored the correlation between *WS* occurrence and various factors, including season, breed, and age. Specifically, we identified a stronger influence of *WS* during Autumn and Winter, among Poodle and Bulldog breeds, and in dogs aged 3 to 10 years on hemorheology. When the temperature decreases, the viscosity of whole blood and plasma increases [26]. Temperature plays a crucial role in blood viscosity; for instance, prolonged immersion in cold water can increase blood viscosity by 19% [27]. Eckmann DM’s study revealed that a decrease in temperature from 37 °C to 18 °C resulted in a 1.2 to 3.5-fold increase in blood viscosity. Similarly, a drop to 15 °C and 0 °C led to increases of 1.8 to 5-fold and 4 to 10-fold, respectively [28]. During the study of giant pandas, it was observed that red blood cell aggregation is at its lowest during the Summer months and peaks during the Winter [29]. In TCVM, cold stagnation can slow down the flow of blood and increase its viscosity. In summary, *WS* is more likely to occur in cold seasons.

Although there is limited research on blood viscosity in dogs, severe brachycephalic obstructive airway syndrome has been shown to be associated with hypercoagulability in dogs [30]. Since Bulldogs are a brachycephalic breed, their blood viscosity is expected to be higher compared to other breeds, and even more so after the occurrence of *WS*. 

In the study of pandas, age was not found to significantly influence hemorheology; however, analysis of average values revealed that ŋp was highest in older individuals, while the EAI, ERI, and EDI were lowest [29]. Research by Racine ML indicates that the EDI is lower in the elderly compared to younger individuals [31]. Somogyi V’s research also showed a decreasing trend in the deformability of red blood cells [23]. Gudmundsson M’s research indicated that fibrinogen concentration increases with age [32]. In our study, the hemorheological indicators of the *WS* groups exhibited a trend consistent with these findings, although the underlying mechanisms require further exploration.

Our research has provided some valuable insights into the connection between *WS* and hemorheology. However, due to limited data, further investigation is necessary to explore more detailed information between to the *WS* and hemorheology, which would help enhance the understanding potential pathogenesis of *WS* from the perspective of hemorheology.

## 5. Conclusions

The study revealed that *WS* in dogs is more prevalent during the cold seasons of Autumn and Winter, and it exhibits a higher occurrence rate in males than females. The breeds with the highest incidence were found to be Poodles, Bulldogs, and mixed breed dogs, primarily affecting dogs between the ages of 3 to 6 years. Importantly, the hemorheological alterations observed in dogs with *WS* were markedly in line with the epidemiological features of this condition, indicating a close association between hemorheology and the onset and progression of *WS*. These findings provide a strategy for the diagnosis and treatment of canine *WS* by considering the specific characteristics of hemorheology.

## Figures and Tables

**Table 1:** Characteristic changes of hemorheology in canine *Wei Syndrome*.

Items	Control (*n* = 53)	*Wei Syndrome* (*n* = 53)
Hŋb 200·S^−1^ (mPa·s)	5.01 ± 0.43	5.59 ± 0.89 *
Mŋb 30·S^−1^ (mPa·s)	5.88 ± 0.55	6.63 ± 1.14 *
Lŋb 1·S^−1^ (mPa·s)	12.51 ± 1.32	14.16 ± 2.79 *
Hŋr 200·S^−1^ (mPa·s)	4.53 ± 1.14	6.74 ± 1.37 *
Mŋr 30·S^−1^ (mPa·s)	5.73 ± 1.35	8.41 ± 1.69 *
Lŋr 1·S^−1^ (mPa·s)	14.91 ± 2.90	20.72 ± 3.82 *
HFR (dyn·s/cm^5^)	34.97 ± 2.99	39.04 ± 6.19 *
MFR (dyn·s/cm^5^)	41.07 ± 3.87	46.31 ± 7.98 *
LFR (dyn·s/cm^5^)	52.56 ± 5.56	60.03 ± 11.48 *
ŋp (mPa·s)	1.78 ± 0.32	1.42 ± 0.20 *
Fb (g/L)	3.83 ± 0.80	2.77 ± 0.66 *
QwX (mPa·s)	3.33 ± 0.29	3.72 ± 0.59 *
CS (dyn/cm^2^)	0.07 ± 0.01	0.08 ± 0.01 *
EDI	0.82 ± 0.10	0.95 ± 0.08 *
EAI	1.89 ± 0.03	1.92 ± 0.06 *
VAI	0.89 ± 0.03	0.92 ± 0.06 *
ERI	4.53 ± 1.14	6.74 ± 1.37 *
Hct (%)	0.42 ± 0.02	0.44 ± 0.03 *

Note: * There is a significant difference compared to the control group (*p* < 0.05). Abbreviations in the table: CS: Carson yield stress; EAI: erythrocyte aggregation index; EDI: erythrocyte deformation index; ERI: erythrocyte rigidity index; Fb: fibrinogen; MFR: medium shear flow resistance; Hct: hematocrit; HFR: high shear flow resistance; Hŋb 200·S^−1^: whole blood high shear viscosity at 200·S^−1^; Hŋr 200·S^−1^: whole blood high shear reduced viscosity at 200·S^−1^; LFR: low shear flow resistance; Lŋb 1·S^−1^: whole blood low shear viscosity at 1·S^−1^; Lŋr 1·S^−1^: whole blood low shear reduced viscosity at 1·S^−1^; Mŋb 30·S^−1^: whole blood medium shear viscosity at 30·S^−1^; Mŋr 30·S^−1^: whole blood medium shear reduced viscosity at 30·S^−1^; QwX: Carson viscosity; VAI: erythrocyte aggregation coefficient; ŋp: plasma viscosity.

**Table 2:** Gender differences in hemorheology of *Wei Syndrome* dogs.

Items	Male		Female	
	Control (*n* = 41)	*Wei Syndrome* (*n* = 41)	Control (*n* = 12)	*Wei Syndrome* (*n* = 12)
Hŋb 200·S^−1^ (mPa·s)	5.01 ± 0.46	5.57 ± 0.85 *	4.98 ± 0.33	5.66 ± 1.04
Mŋb 30·S^−1^ (mPa·s)	5.89 ± 0.59	6.60 ± 1.09 *	5.84 ± 0.43	6.72 ± 1.35
Lŋb 1·S^−1^ (mPa·s)	12.54 ± 1.41	14.04 ± 2.67 *	12.42 ± 1.03	14.56 ± 3.24 *
Hŋr 200·S^−1^ (mPa·s)	4.34 ± 1.08	6.63 ± 1.30 *	5.17 ± 1.15	7.15 ± 1.58 *
Mŋr 30·S^−1^ (mPa·s)	5.51 ± 1.28	8.27 ± 1.61 *	6.49 ± 1.36	8.90 ± 1.95 *
Lŋr 1·S^−1^ (mPa·s)	14.44 ± 2.77	20.38 ± 3.62 *	16.50 ± 2.88	21.87 ± 4.41 *
HFR (dyn·s/cm^5^)	35.03 ± 3.18	38.90 ± 5.93 *	34.77 ± 2.32	39.52 ± 7.29
MFR (dyn·s/cm^5^)	41.14 ± 4.12	46.13 ± 7.64 *	40.81 ± 3.01	46.95 ± 9.41
LFR (dyn·s/cm^5^)	52.67 ± 5.92	59.71 ± 10.95 *	52.17 ± 4.32	61.13 ± 13.59 *
ŋp (mPa·s)	1.83 ± 0.32	1.43 ± 0.19 *	1.61 ± 0.25	1.38 ± 0.23 *
Fb (g/L)	3.92 ± 0.76	2.79 ± 0.63 *	3.53 ± 0.89	2.71 ± 0.78 *
QwX (mPa·s)	3.34 ± 0.30	3.71 ± 0.57 *	3.31 ± 0.22	3.77 ± 0.70
CS (dyn/cm^2^)	0.07 ± 0.01	0.08 ± 0.01 *	0.07 ± 0.00	0.08 ± 0.02
EDI	0.80 ± 0.10	0.94 ± 0.07 *	0.88 ± 0.09	0.97 ± 0.09 *
EAI	1.89 ± 0.03	1.92 ± 0.05 *	1.89 ± 0.03	1.92 ± 0.07
VAI	0.89 ± 0.03	0.92 ± 0.05 *	0.89 ± 0.03	0.92 ± 0.07
ERI	4.34 ± 1.08	6.63 ± 1.30 *	5.17 ± 1.15	7.15 ± 1.58 *
Hct (%)	0.42 ± 0.02	0.44 ± 0.03 *	0.42 ± 0.01	0.44 ± 0.03 *

Note: * There is a significant difference compared to the control group (*p* < 0.05). Abbreviations in the table: CS: Carson yield stress; EAI: erythrocyte aggregation index; EDI: erythrocyte deformation index; ERI: erythrocyte rigidity index; Fb: fibrinogen; MFR: medium shear flow resistance; Hct: hematocrit; HFR: high shear flow resistance; Hŋb 200·S^−1^: whole blood high shear viscosity at 200·S^−1^; Hŋr 200·S^−1^: whole blood high shear reduced viscosity at 200·S^−1^; LFR: low shear flow resistance; Lŋb 1·S^−1^: whole blood low shear viscosity at 1·S^−1^; Lŋr 1·S^−1^: whole blood low shear reduced viscosity at 1·S^−1^; Mŋb 30·S^−1^: whole blood medium shear viscosity at 30·S^−1^; Mŋr 30·S^−1^: whole blood medium shear reduced viscosity at 30·S^−1^; QwX: Carson viscosity; VAI: erythrocyte aggregation coefficient; ŋp: plasma viscosity.

**Table 3:** Seasonal differences of hemorheology in *Wei Syndrome* dogs.

Items	Control (*n* = 53)	*Wei Syndrome*			
		Spring (*n* = 10)	Summer (*n* = 12)	Autumn (*n* = 13)	Winter (*n* = 18)
Hŋb 200·S^−1^ (mPa·s)	5.01 ± 0.43	5.53 ± 0.82	5.33 ± 0.74	6.08 ± 1.14 *	5.44 ± 0.71 *
Mŋb 30·S^−1^ (mPa·s)	5.88 ± 0.55	6.55 ± 1.07	6.29 ± 0.96	7.26 ± 1.47 *	6.44 ± 0.92 *
Lŋb 1·S^−1^ (mPa·s)	12.51 ± 1.32	14.13 ± 2.56	13.46 ± 2.27	15.26 ± 3.93 *	13.84 ± 2.16 *
Hŋr 200·S^−1^ (mPa·s)	4.53 ± 1.14	6.72 ± 1.38 *	6.76 ± 0.91 *	7.16 ± 1.57 *	6.45 ± 1.50 *
Mŋr 30·S^−1^ (mPa·s)	5.73 ± 1.35	8.39 ± 1.72 *	8.38 ± 1.10 *	8.96 ± 1.96 *	8.04 ± 1.83 *
Lŋr 1·S^−1^ (mPa·s)	14.91 ± 2.90	20.73 ± 3.92 *	20.54 ± 2.40 *	22.05 ± 4.52 *	19.88 ± 4.02 *
HFR (dyn·s/cm^5^)	34.97 ± 2.99	38.61 ± 5.76	37.21 ± 5.19	42.48 ± 7.98 *	38.01 ± 4.97 *
MFR (dyn·s/cm^5^)	41.07 ± 3.87	45.78 ± 7.44	43.93 ± 6.67	50.75 ± 10.29 *	45.00 ± 6.40 *
LFR (dyn·s/cm^5^)	52.56 ± 5.56	59.33 ± 10.75	56.53 ± 9.54	66.47 ± 14.87 *	58.11 ± 9.06 *
ŋp (mPa·s)	1.78 ± 0.32	1.41 ± 0.14 *	1.37 ± 0.19 *	1.43 ± 0.21 *	1.44 ± 0.23 *
Fb (g/L)	3.83 ± 0.80	2.84 ± 0.46 *	2.56 ± 0.74 *	2.81 ± 0.64 *	2.84 ± 0.74 *
QwX (mPa·s)	3.33 ± 0.29	3.68 ± 0.55	3.55 ± 0.50	4.05 ± 0.76 *	3.62 ± 0.47 *
CS (dyn/cm^2^)	0.07 ± 0.01	0.08 ± 0.01 *	0.07 ± 0.01	0.08 ± 0.02 *	0.07 ± 0.01 *
EDI	0.82 ± 0.10	0.96 ± 0.07 *	0.97 ± 0.07 *	0.95 ± 0.08 *	0.94 ± 0.09 *
EAI	1.89 ± 0.03	1.92 ± 0.06	1.90 ± 0.05	1.94 ± 0.07 *	1.91 ± 0.05 *
VAI	0.89 ± 0.03	0.92 ± 0.06	0.90 ± 0.05	0.94 ± 0.07 *	0.91 ± 0.05 *
ERI	4.53 ± 1.14	6.72 ± 1.38 *	6.76 ± 0.91 *	7.16 ± 1.57 *	6.45 ± 1.50 *
Hct (%)	0.42 ± 0.02	0.44 ± 0.03 *	0.44 ± 0.03 *	0.46 ± 0.04 *	0.44 ± 0.03 *

Note: * There is a significant difference compared to the control group (*p* < 0.05). Abbreviations in the table: CS: Carson yield stress; EAI: erythrocyte aggregation index; EDI: erythrocyte deformation index; ERI: erythrocyte rigidity index; Fb: fibrinogen; MFR: medium shear flow resistance; Hct: hematocrit; HFR: high shear flow resistance; Hŋb 200·S^−1^: whole blood high shear viscosity at 200·S^−1^; Hŋr 200·S^−1^: whole blood high shear reduced viscosity at 200·S^−1^; LFR: low shear flow resistance; Lŋb 1·S^−1^: whole blood low shear viscosity at 1·S^−1^; Lŋr 1·S^−1^: whole blood low shear reduced viscosity at 1·S^−1^; Mŋb 30·S^−1^: whole blood medium shear viscosity at 30·S^−1^; Mŋr 30·S^−1^: whole blood medium shear reduced viscosity at 30·S^−1^; QwX: Carson viscosity; VAI: erythrocyte aggregation coefficient; ŋp: plasma viscosity.

**Table 4:** Breed differences of hemorheology in *Wei Syndrome* dogs.

Items	Control (*n* = 53)	*Wei Syndrome*		
		Poodle (*n* = 23)	Bulldog (*n* = 8)	Mixed Breed (*n* = 5)
Hŋb 200·S^−1^ (mPa·s)	5.01 ± 0.43	5.67 ± 0.68 *	6.31 ± 1.01 *	5.62 ± 0.72
Mŋb 30·S^−1^ (mPa·s)	5.88 ± 0.55	6.74 ± 0.88 *	7.57 ± 1.30 *	6.67 ± 0.93
Lŋb 1·S^−1^ (mPa·s)	12.51 ± 1.32	14.56 ± 2.12 *	16.54 ± 3.14 *	14.34 ± 2.24
Hŋr 200·S^−1^ (mPa·s)	4.53 ± 1.14	6.89 ± 1.33 *	7.77 ± 1.62 *	6.10 ± 0.77 *
Mŋr 30·S^−1^ (mPa·s)	5.73 ± 1.35	8.60 ± 1.61 *	9.73 ± 2.02 *	7.66 ± 0.97 *
Lŋr 1·S^−1^ (mPa·s)	14.91 ± 2.90	21.19 ± 3.58 *	23.77 ± 4.68 *	19.08 ± 2.20 *
HFR (dyn·s/cm^5^)	34.97 ± 2.99	39.62 ± 4.78 *	44.10 ± 7.06 *	39.27 ± 5.00
MFR (dyn·s/cm^5^)	41.07 ± 3.87	47.07 ± 6.16 *	52.85 ± 9.11 *	46.58 ± 6.47
LFR (dyn·s/cm^5^)	52.56 ± 5.56	61.17 ± 8.89 *	69.47 ± 13.19 *	60.22 ± 9.40
ŋp (mPa·s)	1.78 ± 0.32	1.42 ± 0.22 *	1.37 ± 0.22 *	1.53 ± 0.11 *
Fb (g/L)	3.83 ± 0.80	2.77 ± 0.68 *	2.66 ± 0.84 *	3.19 ± 0.39 *
QwX (mPa·s)	3.33 ± 0.29	3.78 ± 0.46 *	4.21 ± 0.68 *	3.74 ± 0.48
CS (dyn/cm^2^)	0.07 ± 0.01	0.08 ± 0.01 *	0.09 ± 0.02 *	0.08 ± 0.01 *
EDI	0.82 ± 0.10	0.96 ± 0.08 *	0.97 ± 0.08 *	0.92 ± 0.04 *
EAI	1.89 ± 0.03	1.93 ± 0.04 *	1.96 ± 0.05 *	1.92 ± 0.05 *
VAI	0.89 ± 0.03	0.93 ± 0.04 *	0.96 ± 0.05 *	0.92 ± 0.05 *
ERI	4.53 ± 1.14	6.89 ± 1.33 *	7.77 ± 1.62 *	6.10 ± 0.77 *
Hct (%)	0.42 ± 0.02	0.44 ± 0.03 *	0.47 ± 0.03 *	0.44 ± 0.04

Note: * There is a significant difference compared to the control group (*p* < 0.05). Abbreviations in the table: CS: Carson yield stress; EAI: erythrocyte aggregation index; EDI: erythrocyte deformation index; ERI: erythrocyte rigidity index; Fb: fibrinogen; MFR: medium shear flow resistance; Hct: hematocrit; HFR: high shear flow resistance; Hŋb 200·S^−1^: whole blood high shear viscosity at 200·S^−1^; Hŋr 200·S^−1^: whole blood high shear reduced viscosity at 200·S^−1^; LFR: low shear flow resistance; Lŋb 1·S^−1^: whole blood low shear viscosity at 1·S^−1^; Lŋr 1·S^−1^: whole blood low shear reduced viscosity at 1·S^−1^; Mŋb 30·S^−1^: whole blood medium shear viscosity at 30·S^−1^; Mŋr 30·S^−1^: whole blood medium shear reduced viscosity at 30·S^−1^; QwX: Carson viscosity; VAI: erythrocyte aggregation coefficient; ŋp: plasma viscosity.

**Table 5:** Age difference of hemorheology in *Wei Syndrome* dogs.

Items	Control (*n* = 53)	*Wei Syndrome* (Years)			
		0–2 (*n* = 6)	3–6 (*n* = 29)	7–10 (*n* = 12)	11–14 (*n* = 6)
Hŋb 200·S^−1^ (mPa·s)	5.01 ± 0.43	5.89 ± 0.98	5.57 ± 0.92 *	5.71 ± 0.72 *	5.15 ± 0.99
Mŋb 30·S^−1^ (mPa·s)	5.88 ± 0.55	7.02 ± 1.27	6.60 ± 1.18 *	6.78 ± 0.93 *	6.07 ± 1.27
Lŋb 1·S^−1^ (mPa·s)	12.51 ± 1.32	15.21 ± 3.01	13.97 ± 2.93 *	14.64 ± 2.20 *	13.02 ± 3.05
Hŋr 200·S^−1^(mPa·s)	4.53 ± 1.14	7.31 ± 1.35 *	7.06 ± 1.28 *	6.31 ± 1.08 *	5.50 ± 1.63
Mŋr 30·S^−1^ (mPa·s)	5.73 ± 1.35	9.11 ± 1.67 *	8.78 ± 1.60 *	7.92 ± 1.31 *	6.89 ± 2.03
Lŋr 1·S^−1^ (mPa·s)	14.91 ± 2.90	22.27 ± 3.76 *	21.52 ± 3.67 *	19.66 ± 2.90 *	17.45 ± 4.63
HFR (dyn·s/cm^5^)	34.97 ± 2.99	41.14 ± 6.86	38.90 ± 6.42 *	39.86 ± 5.02 *	35.99 ± 6.88
MFR (dyn·s/cm^5^)	41.07 ± 3.87	49.01 ± 8.84	46.12 ± 8.27 *	47.38 ± 6.48 *	42.41 ± 8.87
LFR (dyn·s/cm^5^)	52.56 ± 5.56	63.86 ± 12.65	59.74 ± 11.95 *	61.50 ± 9.25 *	54.70 ± 12.79
ŋp (mPa·s)	1.78 ± 0.32	1.38 ± 0.25 *	1.36 ± 0.16 *	1.50 ± 0.18 *	1.56 ± 0.29
Fb (g/L)	3.83 ± 0.80	2.64 ± 0.98 *	2.60 ± 0.51 *	3.01 ± 0.63 *	3.21 ± 0.87
QwX (mPa·s)	3.33 ± 0.29	3.92 ± 0.66	3.71 ± 0.61 *	3.80 ± 0.48 *	3.43 ± 0.66
CS (dyn/cm^2^)	0.07 ± 0.01	0.08 ± 0.01 *	0.08 ± 0.01 *	0.08 ± 0.01 *	0.07 ± 0.01
EDI	0.82 ± 0.10	0.97 ± 0.09 *	0.97 ± 0.07 *	0.92 ± 0.07 *	0.88 ± 0.10
EAI	1.89 ± 0.03	1.93 ± 0.06 *	1.92 ± 0.05 *	1.93 ± 0.05*	1.89 ± 0.08
VAI	0.89 ± 0.03	0.93 ± 0.06 *	0.92 ± 0.05 *	0.93 ± 0.05 *	0.89 ± 0.08
ERI	4.53 ± 1.14	7.31 ± 1.35 *	7.06 ± 1.28 *	6.31 ± 1.08 *	5.50 ± 1.63
Hct (%)	0.42 ± 0.02	0.46 ± 0.04 *	0.44 ± 0.03 *	0.45 ± 0.03 *	0.43 ± 0.04

Note: * There is a significant difference compared to the control group (*p* < 0.05). Abbreviations in the table: CS: Carson yield stress; EAI: erythrocyte aggregation index; EDI: erythrocyte deformation index; ERI: erythrocyte rigidity index; Fb: fibrinogen; MFR: medium shear flow resistance; Hct: hematocrit; HFR: high shear flow resistance; Hŋb 200·S^−1^: whole blood high shear viscosity at 200·S^−1^; Hŋr 200·S^−1^: whole blood high shear reduced viscosity at 200·S^−1^; LFR: low shear flow resistance; Lŋb 1·S^−1^: whole blood low shear viscosity at 1·S^−1^; Lŋr 1·S^−1^: whole blood low shear reduced viscosity at 1·S^−1^; Mŋb 30·S^−1^: whole blood medium shear viscosity at 30·S^−1^; Mŋr 30·S^−1^: whole blood medium shear reduced viscosity at 30·S^−1^; QwX: Carson viscosity; VAI: erythrocyte aggregation coefficient; ŋp: plasma viscosity.

## Data Availability

The data presented in this study are available from the corresponding authors upon reasonable request.
